# Silk Fibroin: An Ancient Material for Repairing the Injured Nervous System

**DOI:** 10.3390/pharmaceutics13030429

**Published:** 2021-03-23

**Authors:** Mahdi Yonesi, Mario Garcia-Nieto, Gustavo V. Guinea, Fivos Panetsos, José Pérez-Rigueiro, Daniel González-Nieto

**Affiliations:** 1 Center for Biomedical Technology, Universidad Politécnica de Madrid, 28223 Pozuelo de Alarcón, Spain; mahdi.yonesi@ctb.upm.es (M.Y.); gustavovictor.guinea@ctb.upm.es (G.V.G.); 2 Silk Biomed SL, 28260 Madrid, Spain; fivos@ucm.es; 3 Cruz Roja Española, 28003 Madrid, Spain; mario.garcia@cruzroja.es; 4 Departamento de Ciencia de Materiales, ETSI Caminos, Canales y Puertos, Universidad Politécnica de Madrid, 28040 Madrid, Spain; 5 Biomedical Research Networking Center in Bioengineering Biomaterials and Nanomedicine (CIBER-BBN), 28029 Madrid, Spain; 6 Neurocomputing and Neurorobotics Research Group, Faculty of Biology and Faculty of Optics, Universidad Complutense de Madrid, 28040 Madrid, Spain; 7 Innovation Group, Institute for Health Research San Carlos Clinical Hospital (IdISSC), 28040 Madrid, Spain; 8 Departamento de Tecnología Fotónica y Bioingeniería, ETSI Telecomunicaciones, Universidad Politécnica de Madrid, 28040 Madrid, Spain

**Keywords:** silk, biomaterials, polymers, stem cells, drug delivery, neurological disorders, stroke, Alzheimer, peripheral nerve injury

## Abstract

Silk refers to a family of natural fibers spun by several species of invertebrates such as spiders and silkworms. In particular, silkworm silk, the silk spun by *Bombyx mori* larvae, has been primarily used in the textile industry and in clinical settings as a main component of sutures for tissue repairing and wound ligation. The biocompatibility, remarkable mechanical performance, controllable degradation, and the possibility of producing silk-based materials in several formats, have laid the basic principles that have triggered and extended the use of this material in regenerative medicine. The field of neural soft tissue engineering is not an exception, as it has taken advantage of the properties of silk to promote neuronal growth and nerve guidance. In addition, silk has notable intrinsic properties and the by-products derived from its degradation show anti-inflammatory and antioxidant properties. Finally, this material can be employed for the controlled release of factors and drugs, as well as for the encapsulation and implantation of exogenous stem and progenitor cells with therapeutic capacity. In this article, we review the state of the art on manufacturing methodologies and properties of fiber-based and non-fiber-based formats, as well as the application of silk-based biomaterials to neuroprotect and regenerate the damaged nervous system. We review previous studies that strategically have used silk to enhance therapeutics dealing with highly prevalent central and peripheral disorders such as stroke, Alzheimer’s disease, Parkinson’s disease, and peripheral trauma. Finally, we discuss previous research focused on the modification of this biomaterial, through biofunctionalization techniques and/or the creation of novel composite formulations, that aim to transform silk, beyond its natural performance, into more efficient silk-based-polymers towards the clinical arena of neuroprotection and regeneration in nervous system diseases.

## 1. Introduction

The nervous system is composed of specialized cells organized in complex networks with the ability to integrate and adopt signals from different tissues and organs. The nervous system receives sensorial inputs from the outside environment and sends signals to the periphery cells and muscles to execute simple and complex motor commands [1]. In the brain, variable distribution of neural networks that have not yet been completely deciphered supports cognitive abilities such as verbal, visual learning, and memory. Like other tissues the neural tissue is subjective to senescence, degeneration, and occasional damage. However, the nervous system shows a poor regeneration potential, which is still a motive of controversy [2,3]. Many efforts of regenerative medicine focus on providing “fresh” cells to structurally and functionally replace neural cells lost after degeneration or trauma. Stem cell (SC) delivery is a popular therapy due to their pluripotency and multipotency ability for direct differentiation into neural lineages and/or the release of specific factors that can stimulate endogenous neurogenesis and self-repairing mechanisms [4,5,6]. For example, the implantation of Embryonic Stem Cells (ESC) and Neural Stem Cells (NSC) have been used in very different contexts such as spinal cord injury [7,8] or Parkinson’s disease [9,10]. Mesenchymal stem cells (MSC) are also commonly used since they can overcome specific limitations usually found with other stem cell cells; namely the difficulty of isolation and expansion and ethical and safety (i.e., tumorigenesis) issues [11,12,13]. To restore the functionality lost, the transplanted cells should not be just mere spectators in the evolution of the pathological process, and their differentiated progeny should be able to structurally and functionally integrate with the remaining non-damaged host tissue.

However, cell therapy is faced with the strong decline in survival observed for the majority of transplanted cells. This fact is even more accentuated in the nervous tissue, a very hostile environment for donor cells, independently of their germinal origin. For example, MSC show poor survival after brain transplantation into the brain as soon as one week post-transplantation [14]. MSC viability is even lower in the injured brain [15]. Similar circumstances occur with other stem cell phenotypes. For example, NSC showed reduced survival after transplantation in the ischemic brain, although NSC survival and in vivo expansion can be stimulated by mitogenic factors [16]. Due to these limitations, current research aims to develop strategies for increasing cell content and engraftment. However, grafted cell content must be strictly controlled, since beyond therapeutics, excessive in vivo expansion might lead to uncontrolled division and tumor formation. An interesting opportunity in this context emerges from the use of matrices (or scaffolds) to favor cell engraftment in specific desired locations. In addition to cells, the direct delivery of stimulating factors from these scaffolds is also possible. Suitable scaffolds should satisfy several criteria such as appropriate bioactivity, biomechanical properties, and biocompatibility to mimic the anatomical and physiological environment of the target tissue [17].

**Figure 1:**
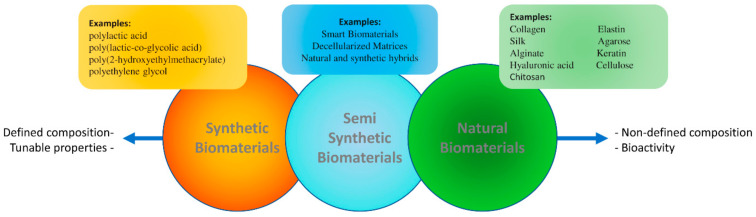
Biomaterials are divided into synthetic, natural, and semi-synthetic categories. While the properties of synthetic biomaterials include a defined composition and tunable mechanical and chemical properties, natural biomaterials are characterized by a less-defined composition, but show an inherent bioactivity due to the presence of natural extracellular matrix motifs. Thus, adequately selected and processed natural biomaterials tend to show higher biocompatibility when compared with their artificial counterparts.

Matrices for tissue engineering have a diverse origin (Figure 1). Natural matrices are obtained from extracellular matrix (ECM) components produced by living organisms. Biological materials such as collagen, fibronectin, and laminin can provide molecular cues such as the Arginine-Glycine-Aspartic (RGD) motif and, in addition, provide structural support to the scaffold [18,19,20]. In contrast, their structure and reproducibility are not as well defined as that of synthetic biomaterials, an artificial route to mimic ECM properties [18,21]. Thus, synthetic biomaterials such as polycaprolactone (PCL) and poly-D, L-lactide (PLA), provide a well-defined structure at different levels of observation with controllable and repetitive properties, though showing limited biocompatibility [22,23]. The lack of biological sequences in synthetic biomaterials is a challenge that can be partially addressed by further chemical or physical modifications. Some studies have reported the drawbacks of these approaches, such as those relying on graphene [24] or carbon nanotubes [23].

In an attempt to combine the advantages of natural and synthetic sources ECM-derived matrices have been proposed [18,25]. ECM-derived matrices are obtained from the decellularization of organs and tissues and can provide a solution for the accurate reproduction of the anatomical and physiological environment. Nevertheless, several steps in the processing of these scaffolds (i.e., the usage of different solvents) might modify their biomechanical and biological properties. In addition, special attention needs to be paid to the decellularization process and RNA/DNA decontamination, which should be done without changing the biochemical and biomechanical properties of the tissue while removing any trace of cellular and immunogenic material from donated tissues. During decellularization, the biomechanical properties can be impaired and many extracellular matrix proteins and signaling molecules are usually lost [26,27]. A main limitation of matrices based on decellularized tissues is the rate of cell repopulation, which is usually inefficient and random. Additionally, decellularized matrices can be subjective to safety and ethical problems for clinical use [28].

Alternatively, electrospun matrices have been widely applied to tissue engineering for several decades. However, cellular penetration and limitations associated with the toxicity of undesirable by-products and chemical molecules from electrospinning dopes are a serious concern.

Independently of its origin, the properties of a biomaterial can be further improved if the matrix shows the ability to adapt to the cellular environmental and physiological signals. This concept can be turned into reality by the addition of informational domains, by cross-linking the biomaterial and by making natural/synthetic composites [29,30]. In particular, the application of smart biomaterials has been proposed for cell and drug delivery [30,31]. However, the lack of sufficient knowledge about the biological interaction with cells and tissues is a main limitation of this approach. This is particularly evident in the field of neural tissues and represents a non-resolved challenge to be faced in the clinical arena [29], leaving open the search for new biomaterials.

In this context, silk-based biomaterials stand out for its versatility and natural biocompatibility. Although silk is regarded as a natural polymer it shows strong potential as a tunable biomechanical and biochemical material in various formats. Silk biomaterials have been applied, e.g., in bone, cartilage, muscular, vascular, and skin tissue engineering [32]. In recent years, their applicability has moved to the field of neurobiology. The unprecedented potential of silk proteins in providing a regenerative environment for the growth, proliferation, and maturation of various neural cells in allografts and autografts has been demonstrated [32]. Another therapeutic facet of silk biomaterials is related to its ability to deliver bioactive compounds and help to reduce tissue inflammation and oxidative stress and induce cellular proliferation following an efficient rate of biodegradation and the ability to modify the bulk and surface properties of the material [33,34,35]. Silk scaffolds can be fabricated in a variety of formats such as hydrogels, mats, fibers, and films, and this versatility is one of the beneficial characteristics for usage toward various neural diseases.

In this article, we present and discuss previous reports and current trends in the field of using silk biomaterials applied for neural tissue engineering in neurodegenerative diseases and different trauma conditions.

## 2. The Source of Silk Fibroin: From Nature to Laboratory

Silk is probably one of the oldest known natural materials that have accompanied the history of humanity. It was already used in the early Neolithic Age, more than 8500 years ago [36] and nowadays, silk is utilized in a variety of formats for such applications such as drug delivery, tissue engineering, electronics, or optics [37].

Although silk is naturally spun by various species of insects and spiders, studies about silkworm (*Bombyx mori*) silk have attracted most of the attention due to their large availability from controlled and non-expensive sources. Natural silkworm silk is composed of two families of proteins: silk fibroin (SF) and silk sericin (SS). As a result, in this article it is intended to use the word “silk” only as long as the combined presence of SS and SF is implied. SF shows negligible immunogenicity and forms fibers insoluble in water. By contrast, SS is a water-soluble biocompatible protein. Both SS and SF are reported to exhibit various neuroprotective, anti-inflammatory, and anti-oxidant properties. For reasons that are still unclear, the combined use of fibroin and sericin in an organism tends to induce a proinflammatory reaction [38], but natural silk fibers can be applied if conveniently treated to remove the sericin coating [39,40]. Most biomedical applications of silk, however, rely on the usage of regenerated silk, which implies a first step of dissolution of the silk fibroin fibers and the subsequent creation of the intended format (fibers, gels, sponges, etc.). After removing the sericin coating, the natural native silkworm silk is composed of three proteins known as heavy chain (about 391 KDa), light chain (about 26 KDa) and P25 protein (about 25 KDa) [41,42]. Consequently, regenerated silk fibroin solution is composed of these three different proteins, although it is generally assumed that the main properties of the biomaterials produced from these solutions result from the presence of the heavy chain protein. The solution of the fiber leads to a certain degradation of the proteins, in comparison with the native proteins not subjected to this treatment. In compensation, the usage of regenerated silk usually improves biocompatibility due to the purification in the material induced by the process of regeneration and increases the reproducibility of the material.

The silk required for biomedical uses is normally obtained from three sources, depending on the concrete silk type to be used.

1. Forcibly spinning of the fibers from the silk-producing species (mainly spiders) [43,44]. Spider silk fibers are biocompatible, after the removal of the possible contaminants, and are commonly used as guiding biomaterial [44]. Spider silk lacks the sericin coating, which is present in the forcibly silked fibers retrieve from silkworms [45]. However, the usage of this method is complex, cost-intensive, and—most importantly—provides only very small quantities (up to mg), far from the required amounts for the biomedical context outside the laboratory.
2. Extraction of the proteins from the natural source (mainly from the silkworm). This extraction can be done directly from the silk glands of the animal [46,47] or by dissolving the proteins of the spun fibers during the process of degumming and regeneration [48]. This latter method is the most common for tissue engineering applications and comprises the largest proportion of reported research in neural tissue engineering (Table 1), so that it will be the main focus of this review.
3. Production of recombinant silk proteins by cloning the gene expressing the silk fibroin in a host organism [49,50,51]. This approach can be a good answer to provide enough silk biomaterial with tailored properties for clinical usage. Silk genes can be modified to further enhance the quality and tunability of the final biomaterial [51]. However, the recombinant production of silk show limitations due to the different amino acid codon preference of the host organism with respect to the donor insects. This limitation, as well as the possible contamination with pyrogens, becomes more significant in the case of bacterial hosts.

**Figure 2:**
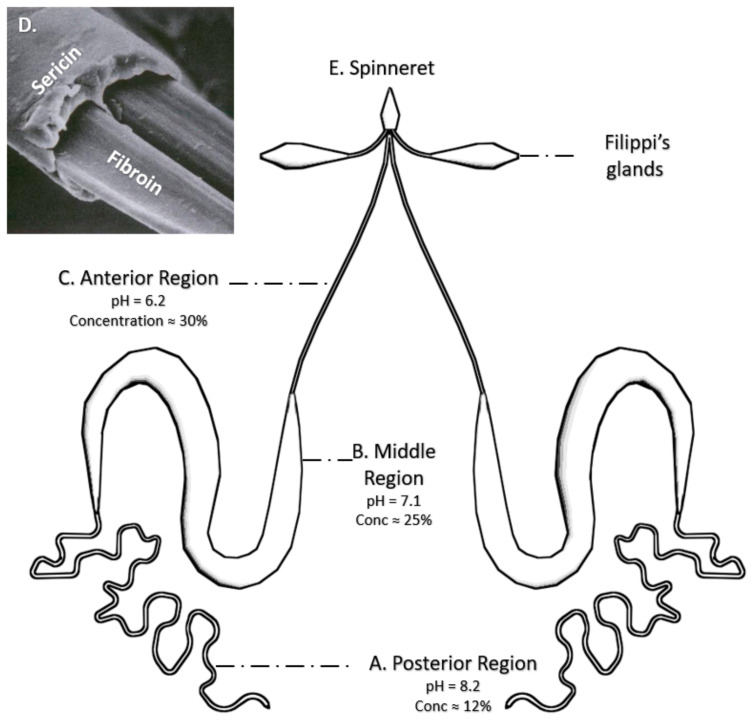
The silk fiber is made within the silk glands of *B. mori*. (**A**) The primary secretion of the silk fibroin occurs in the posterior region of the silk glands, where it reaches a concentration of around 15% and a pH of 8.2. (**B**) The SF solution is concentrated and acidified in the middle region. Sericin is secreted by the middle silk gland cells. (**C**) Finally, the SF enters the anterior part, in which the process of acidification continues until a pH value of 6.2 and a concentration of up 30% are achieved. (**D**) The functional fiber (bave) is composed of two fibroin monofilaments (brins) covered by a sericin layer (SEM image reproduced with permission from [69], MDPI, 2018); (**E)** The fiber is secreted from spinneret for the formation of the silk structure.

Silk fibers are originally produced in the glands of the silkworm [66] (Figure 2) from an aqueous fibroin solution [37]. The formation of the solid fiber results from a combined physicochemical (a decrease in pH and an increase in the protein concentration along the gland) and mechanical (tangential stresses exerted on the solution) action [37,66]. The combined action of both effects leads to the transition from the soluble Silk I conformation to the insoluble, β-pleated sheet-rich, Silk II phase [67]. Silk I and Silk II forms are discussed below.

**Figure 3:**
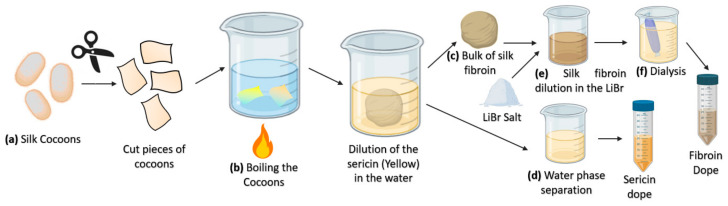
(**a**) The process of sericin extraction from the fibroin in the laboratory (degumming process) is initiated by cutting the cocoons into small pieces. (**b**) Sericin is soluble in water, but fibroin fibers are not. After treatment with water (yielding fibroin with a higher molecular weight) or with the addition of salts, such as sodium carbonate (yielding fibroin with a low molecular weight) the fibroin will form a bulk of fibers in the solution (**c**), and sericin will remain dissolved in the water phase. (**d**) Sericin can be concentrated for other applications. (**e**) The degummed fibroin is dissolved in an aqueous 9.2 M LiBr solution. (**f**) The high concentration of the salt for dissolving the protein is removed by dialysis and a silk fibroin dope is achieved for the subsequent applications.

The resulting silk fiber (bave) is composed of two fibroin monofilaments (brins) surrounded by a sticky sericin layer [66] that flues the fibers to form the cocoon. Consequently, extracting SF in the laboratory requires to dissolve the hydrophilic sericin protein in water—a process called degumming—preferably with heat and mild acidic or alkaline solution (such as sodium carbonate) and/or with the addition of detergents (Figure 3). The degumming process ordinarily degrades the fibers and results in an SF with a molecular weight lower than the natural one. A higher molecular weight can be obtained by employing water without additives. As SS represents about 25% of all fibers in the *B. mori* cocoons, this amount of decrease in the net weight is expected after completing the degumming process [48]. A SF solution is usually obtained by dissolving the degummed fibroin fibers in a 9.2 M LiBr solution in water. The excess of salt is removed with one or several dialysis steps, leading to the production of a silk protein solution called dope that can later be used directly in liquid form or as a lyophilized dried powder [68] (Figure 3).

## 3. Fabrication Methods and Platforms for Regenerated SF-Based Biomaterials

Several procedures have been described to obtain the protein solution require for the creation of the different regenerated silk fibroin formats. For example, Zhous et al. (2019) used the solution prepared by mechanically stirring the silkworm cocoon in an alcoholic solution [70]. However, such methods provide a lower control on the properties of the solution since, for instance, the protein concentration is not easily measured. By far, however, most methods that use regenerated silk fibroin follow a series of common initial steps as summarized in Figure 3.

As indicated above, the secondary structure and the molecular assembly of SF are important features not only for the formation of the native fibers, but also with regard to the fabrication of the 2D and 3D scaffolds for tissue engineering. In this respect, the presence of β-sheet crystalline structures that lead to water insolubility is most relevant. Shimizu (1941) reported for the first time that silk proteins are found in two forms, which later were called Silk I (soluble) and Silk II (insoluble) [71,72]. A further instable Silk III structure was also described, but it is not usually found in SF-based biomaterials. Silk I is a metastable structure formed from silk dope after drying or being concentrated [73]. This form is composed of metastable β-turns, S zigzags, and crankshaft conformations as well as α-helices and random coils [74]. Silk I can be found in SF films and leads to inferior mechanical properties and stability in an aqueous solution [73]. SF films performance can be improved by the utilization of SF in a high concentration and slow drying protocol [74].

Silk II appears as a consequence of the tendency of the Gly-Ala-Gly-Ala-Gly-Ser motifs to arrange into a stable anti-parallel β-sheet conformation [74]. The presence of β-sheets increases the strength and stability of silk biomaterials, especially in an aqueous solution. In the process of biomaterial fabrication, the conversion of non-β-sheet content into an arranged β-sheet composition has been attempted to confer stability and strength to the SF biomaterial. The Silk II structure can be obtained from the transformation of Silk I upon extreme heat, vibration, electronic stimulation, the usage of organic solvents (such as ethanol and methanol), vapor annealing or slow drying [75]. In the field of neural tissue engineering, biomaterials with Silk II structures are preferred because of their higher stability in an aqueous solution, although some Silk I content might be useful for controlling the degradation properties of the material. It is assumed, however, that an increase in the proportion of Silk II phase increases the stability of the material and might be beneficial for increasing it shelf-life. As a result, SF biomaterials with a sufficient of Silk II conformation can be shaped into a variety of 2D and 3D geometries (such as hydrogels, fibers, films, and meshes) with tunable physical properties.

**Figure 4:**
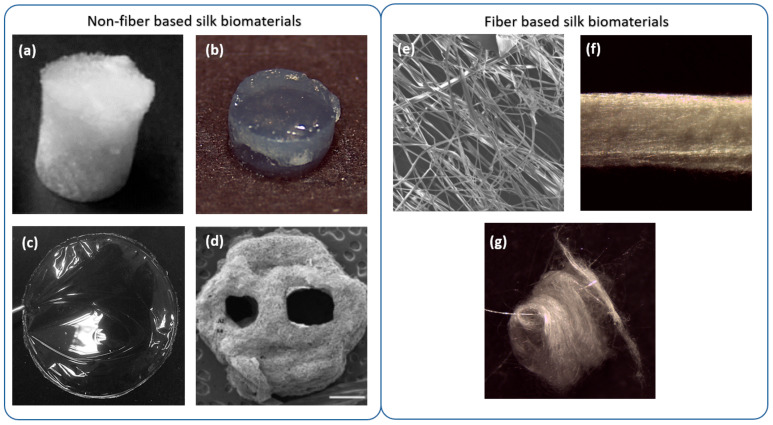
SF formats are divided into fiber and non-fiber-based formats. Non-fiber-based formats are composed of crosslinked fibroin molecules such as (**a**) silk sponges (image adapted with permission from [61], ACS publications, 2015); (**b**) hydrogels, (**c**) films and (**d**) 3D-printing patterns with or without polarity and direction (image adapted with permission from [57], Neural Regeneration Research, 2020) Fiber based biomaterials are composed by fibers of different types that can be (or not) arranged in a defined direction. Fiber based biomaterials include (**e**) fibers, (**f**) bundles, and (**g**) mats (or non-wovens).

In this review, we have divided the strategies for the fabrication of silk-based biomaterials into fiber and non-fiber-based formats as illustrated in Figure 4. Table 1 shows a summary of the most common SF biomaterials that have been applied in the field of neural tissue engineering with a summary of their fabrication methods.

### 3.1. Fabrication of Non-Fiber-Based SF Biomaterials

Silk regeneration from silk dopes is the process that induces the production of highly arranged and stable Silk II structures in the biomaterial, making the biomaterial insoluble in water and with appropriate stiffness and strength [76]. The regeneration is based on the potential of SF to form β-sheet nanocrystals upon stimulations with several physical-chemical agents, such as changes in pH (acidic), temperature, mechanical and shear stress stimulation (vibration and sonication), osmolarity changes or the addition of cross-linking agents (Figure 4). Biomaterials resulting from silk regeneration can be used through different SF formats, which are discussed below.

#### 3.1.1. Sponges

Silk fibroin sponges (Figure 4a) are produced in various ways, such as lyophilization [61] or by drying the casted silk fibroin solution within an oven [59]. The porosity, initial concentration, and post-processing might affect the mechanical properties of the biomaterial [37]. Biomaterial porosity can be controlled with gas foaming, salt leaching, or automatically with lyophilization methods. The porosity is beneficial, as it provides a matrix for cell homing and for lodging other types of components to further simulate the physiological environment of the tissue. Kaplan and coworkers [61] have assessed fabrication of lyophilized sponges with different concentrations and porosity. It was shown that SF sponges can be used as a versatile and tunable material for soft and hard tissue engineering with high biocompatibility and potential for maintenance of stem cells [61]. Kaplan’s group proposed the integration of collagen type I within the pores of SF sponges, providing a good model of a polarized nervous tissue structure such as the gray matter in the brain [59]. A fabrication method of sponges in a donut configuration as a model of cortical brain tissue was later employed [60]. SF-collagen donut-shape sponges were used for culturing primary cortical neural cells from chick dorsal root ganglion explants. This assessment showed silk sponges as a promising substrate for drug screening/testing and an in vitro model of neuronal damage. SF sponges offer a tunable elasticity modulus that depends on different parameters, such as the degumming time, the degree of crystallinity or the concentration of silk. The elasticity modulus in sponges varies from 50 to 350 kPa [61], displaying higher values than SF hydrogels (4.8–33.1 kPa) [77,78,79], and particularly greater than nervous tissue (below 100 kPa). Consequently, the research trend in SF sponges tends to move to low polymer silk concentrations (<2%) and a further intensive regime of degumming to reduce the mechanical properties [59,60,80].

#### 3.1.2. Silk Hydrogels

Silk hydrogels can be produced from the solution containing the regenerated silk as a consequence of its meta-stability. Changes in temperature, mechanical shear stresses, ultrasound waves, variations in composition, such as modifying pH or extracting/adding salts can lead to the gelation as the result of the self-assembly process of silk fibroin. Strategies such as using vortex agitation [81], sonication [77] and electric field exposal [82] are examples of the technics that have been used to induce the process of self-assembly. Hydrogels are efficient and controllable and can favor cell survival and engraftment within the damaged tissue. The 3D hydrogel structure can preserve and protect the therapeutic cells inside while allowing the entrance of nutrients and oxygen, as well as the expulsion of waste products. Silk hydrogels (Figure 4b) are easily adjustable in terms of mechanical properties and water content, so that the modification of these properties can significantly impact cell growth and function [83]. Control of the biodegradation rate of the silk hydrogel and its high bioactivity makes this biomaterial a choice for soft tissue engineering in comparison to other hydrogel-based formulations, such as polydimethylsiloxane (PDMS) and agarose [84,85], that show a negligible degradation due to the lack, in mammalian tissues, of enzymes able to decompose such materials [86,87]. A tunable degradation rate can ensure enough time for the regeneration of damaged nervous tissue. An important feature of silk hydrogels is the potential of this biomaterial to create a self-assembling injectable platform that can be used for the provision of cells, therapeutic compounds, and biomaterials to the point of injury, after brain or spinal cord damages [77,88,89]. For soft neural tissues, silk hydrogels can improve the expansion of NSC within the biomaterial and present an ideal rate of drug delivery together with tunable mechanical properties and a good rate of biodegradation [78,90]. Our group found that 2% is probably the most appropriate SF concentration for intra-cerebral applications. This concentration produces an elastic modulus that is similar to the host tissue. In addition, this polymer concentration is most suitable for stem cell survival and engraftment [62,78,79,88]. It has been found that SF polymer concentrations greater than 2% considerably reduced the viability of MSC in the interior of SF hydrogels, probably due to an excessive compartmentalization [63]. In addition, this combination of MSC and 2% SF hydrogels induced functional recovery in a stroke mouse model [62], probably due to the anti-inflammatory properties of MSC, which were strongly stimulated after encapsulation [91]. The analysis of SF hydrogels in concentrations higher than 2% has been done in composites with gelatin—glycidyl methacrylate. In this case, MSC encapsulated in SF at 2.3% and 5% demonstrated neurite outgrowth and an increasing expression of brain-derived neurotrophic factor (BDNF) as an indicator of transdifferentiation of MSC to neuronal cell-like phenotypes. In addition, 5% composite hydrogels provided enough mechanical properties for the maintenance of the cells [79].

#### 3.1.3. Silk Films

Silk films (Figure 4c) are fabricated in various ways including spin-casting, dry-casting, and layer-by-layer assembly by spinning [37]. Silk films are well tolerated by neural cells, and no evidence of cell death, inflammatory microglia, or astrocyte activation was reported when silk films were deposited over a cortical brain surface [35]. A limitation of SF films is their brittleness upon regeneration due to the excessive β-sheet content. A solution to provide further flexible films is vapor annealing the silk films, which results in a lower β-sheet content and further organized molecular packing compared to other methods such as dry-casting and alcohol treatment [37]. SF films show considerable electroconductive properties [92], and neuronal axonal growth has been achieved with a combination of SF films, neural cells and electrical stimulation [64]. In one study, SF film-coated electrodes were implanted in the rat brain [65]. Electrical stimulation evoked calcium responses, and SF implants were able to modulate host brain cells with marginal inflammation [65]. Finally, SF films provide a potentiality for loading different growth factors (GFs), drugs, and assessments of other surface modification strategies that will be discussed in subsequent sections. For example, SF films have been applied directly on the cortical brain surface to bypass the blood-brain barrier (BBB), sustaining the delivery of chemotherapy agents against brain tumors. This technological concept was developed by Tang-Schomer and co-workers using drug-loaded SF films with AraC, a mitotic inhibitor, to selectively inhibit glial cancer cells [35]. This way, small molecules delivered from SF films reached deep brain structures as the hippocampus, and no toxicity effects associated with the implanted material were reported [35].

Collectively, non-fiber-based SF biomaterial fabrication methods can produce 2D and 3D biomaterials that can be used as biocompatible grafts to treat neuronal damages. However, these biomaterial configurations normally lack direction and polarity. After SF regeneration, the formed β-sheet does not show any orientation, and the biomaterial itself is not orientated, so it cannot guide cells to a specific path, which is especially important, for example, for rewiring damaged axonal tracts. This limitation might be compensated by surface modification, considering chemical and physical alterations that could enhance or provide patterns for cell growth, which is discussed in the following sections [64,93]. It is also possible to fabricate fibrillar structures of various thicknesses to produce different geometries, with the control of polarity and direction in the biomaterial.

#### 3.1.4. Solid Free Form Formats

Solid Free Form Fabrication (SFF) techniques, also known as Rapid Prototyping techniques, groups a number of processing techniques, that include 3D printing, that create shapes by the addition of subsequent layers of material. SFF techniques and, in particular, 3D printing, allow fabricating silk structures with detailed custom shapes and geometries based on necessity (Figure 4d). There are various methods of 3D printing that are used for different types of materials and different tissue engineering approaches [94]. 3D bioprinting offers the possibility to integrate the cells in a matrix with the potential of supporting the cells during the process of printing [95]. One of the challenges of 3D bioprinting is the design of proper bioinks with suitable mechanical and biocompatible properties [96]. This process has been inspected by a limited number of researchers in the arena of SF [57,58]. Zhao et al. used polypyrrole 3D-printed patterns with electrospun fibers of silk fibroin to further improve the electrochemical properties of the biomaterial [58]. This composite was successful for the arrangement of cells and did not show any toxicity, while presenting good potential as a neuronal conduit in peripheral nervous system injuries [58]. A collagen/SF bioink formulation was 3D-printed by Jiang and coworkers to seed NSC [57]. This combination showed good potential for nervous tissue regeneration after spinal cord injury. There are a limited number of studies in 3D bioprinting using silk for neural applications. Due to the variety of 3D printing methods and the high potential of 3D printing technology to create precise custom shapes, more studies using new silk bioprinted composites for neural tissue engineering are needed. For further information on this topic, several comprehensive reviews are given in the reference section [97,98].

### 3.2. Fabrication of Fiber-Based Biomaterials

Fiber-based SF formats (Figure 4e–g) can be produced by various methods, including dry and wet spinning. SF fibers can be gathered in various collectors to make 2D and 3D highly arranged mats or weaves of different shapes. This method has the potential to fabricate highly arranged structures to guide cells and push them toward further proliferation, differentiation, and adhesion and to be used as conduits for axonal guidance and neuronal regeneration [44,99,100]. A main issue is the hardship of constructing structured 3D scaffolds to fill spatial defects in the nervous tissue [101]. Consequently, directional SF biomaterials such as fibers and bundles are commonly employed to provide conduits to regenerate and rewire nerves, as for example in peripheral nervous system damages or for axonal guidance at a central nervous system level, in disorders where a functional disconnection between specific areas is produced, for example, in Parkinson’s disease. Alternatively, to compensate for the lack of arrangement in non-orientated 3D scaffolds, it is possible to integrate the SF fibers in 3D scaffolds to adjust spatial limits [102]. In the following section, the application of various types of silk formats for both extensive and localized neuronal damages is discussed.

#### 3.2.1. SF Fibers

SF fibers (Figure 4e) can be formed from various techniques, such as electrospinning, dry spinning, wet spinning, and straining flow spinning, depending on the features and necessary diameter for specific applications. Perhaps the most common technique for the fabrication of silk fibers for practical usage is electrospinning, a method that provides fibers with diameters ranging typically from 10 to 1000 nm. Handling such fibers for physical and chemical characterization is mainly done not as individual fibers, but as a bulk of fibers in either orientated (bundle) or non-orientated (mat) patterns. The electrospinning technique induces the regeneration of stable Silk II structures. However, the resulting fibers are not stable in an aqueous solution, due to the partial content of Silk I conformation even after completing the spinning process [54], and very often a post-spinning treatment is needed for stabilization, such as immersion in methanol or formic acid [101]. Mechanical properties of SF fibers are thus dependent on the processing parameters such as SF concentration, collector distance, speed and electrical field as well as on the post-spinning conditions [37]. Electrospinning dopes usually employ organic solvents (such as trifluoroethanol and hexafluoroisopropanol), which are toxic for biomedical applications, and affect the bulk properties of silk fibers and mats [103].

**Figure 5:**
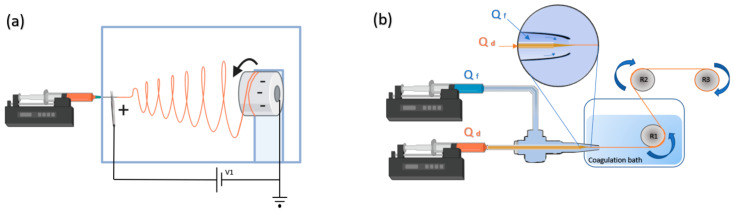
Example of two spinning-based techniques used for the creation of fiber-based SF biomaterials. (**a**) Electrospinning induces the formation of beta-sheets by pulling the dope through the application of an electrical potential and fast evaporation of the solvent. Fibers are gathered in collector (in this case, rotating). Fiber properties are dependent on the injection flow rate, the needle–collector distance, the collector shape, the collector speed, the dope concentration and the voltage (V1) applied to the system. Usually a post-spinning stage (not shown) is needed to stabilize the fibers. (**b**) Straining flow spinning (SFS^®^) proceeds by the combined pulling of the SF dope by means of a coaxially surrounding flux (focusing fluid) and the take-up mandrel. SFS allows the production of high-performance fibers through the control of more than 10 processing parameters, among which are the flow rate of the dope (Qd), the flow rate of the focusing fluid (Qf), and the speed of the take-up mandrel. The dope enters a coagulating bath in which the solidification process is completed. The fiber is initially retrieved in the take-up roller (R1) and may be later subjected to post-spinning drawing treatments with additional mandrels (R2, R3).

To address this problem, our group has developed the biomimetic Straining Flow Spinning (SFS^®^, Heerbrugg, Switzerland) technique to fabricate fibers with tunable mechanical properties. This technique is inspired on the natural spinning process of spiders and silkworms [52]. In this method, SF microstructure is induced by combination of physicochemical changes and mechanical stresses (Figure 5). Individual fibers (from 10 to 100 µm diameter) can reach high performance tensile properties like natural materials. Unlike electrospinning, which results in the fabrication of biomaterials in a partial Silk I state, the SFS method allows for the fabrication of SF structures at the Silk II stage and modifying the conformation of the fibroin proteins in a controlled and predictable way. In addition, SFS leads to the production of fibers without the usage of the aforementioned harsh solvents. Silk fibers produced by SFS have been assessed in vitro and have promoted the organization of cortical primary cells allowing for the structural re-connection of distant neural spheroids, favoring neural cell migration and axonal guidance [53].

#### 3.2.2. Silk Mats

Silk mats (Figure 4g) are tunable and orientated 3D structures of silk fibers that can be fabricated by techniques such as electrospinning, 3D printing, and other custom-made techniques. Mats can be employed for the guidance of neural tissue regeneration. Mats can be generated by the stockpile of 2D bundles and meshes forming, e.g., tubular or planar arrangements. The 2D surfaces provide a high surface area to mass ratio with remarkable mechanical properties [101]. The advantage of this fabrication method is to create highly organized patterns of fibers that mechanically guide seeded cells to regenerate the damage [104]. The mechanical properties of silk mats are a function of the solvent system, the fiber arrangement, and the mechanical properties of the individual fibers themselves. Generally, a more condensed and entangled mat can lead to overall higher mechanical properties. Approaches such as electrospinning provide an ability to fabricate fibers on a similar scale as neural tissues and are capable of producing robust and highly arranged mats and tubes with nanometer inter-fiber distance for further biomimetic tissues [105,106]. Silk mats are found to support cell proliferation and migration of astrocytes followed by neurite outgrowth [56].

## 4. Silk Fibroin to Revert Pathological Conditions of Stroke

The nervous system is composed of the central nervous system (CNS), which comprises neurons and glial cells in brain and spinal cord, and the peripheral nervous system (PNS), mainly formed by nerve bundles that extend in many parts of the body including limbs and organs. Because of the highly specialized function and inter-connected wiring between CNS and PNS, a central or peripheral injury might have dramatic consequences in sensorial, motor, or cognition capacities.

In humans, the most prevalent neurological pathology is ischemic stroke, which is caused by a deficiency in the provision of sufficient blood and oxygen to the brain. Brain stroke might produce vision impairment, loss of cognition, and body limb deficits. The symptoms are variable depending on the affected area.

Stroke produces a core or irreversible injury surrounded by an ischemic penumbra that can be salvable [107]. Inflammation, excitotoxicity by neuronal neurotransmitters, and oxidative stress contribute to transforming the ischemic penumbra into irreversibly damaged tissue [107]. This process occurs during the acute stage of disease (hours to several days after the onset of oxygen deprivation), and multiple molecular and cellular pathological processes contribute to establishing a definitive lesion, reaching a time point when neuroprotective agents are no longer efficient. Extensive damage affects a vast area of neuronal tissue and produces a 3D cavity, causing changes in the biomechanical properties of the tissue and loss of connection between different areas. Neural damage and inflammation, especially driven by microglia and reactive astrocytes, remodel the brain tissue and form a fibrotic scar.

To revert this pathological condition, main neuroprotective strategies are orientated to reduce inflammation, excitotoxicity and/or oxidative stress. Later, once the damage is established (chronic phase), the replacement of neural damaged circuitry and/or the stimulation of endogenous repairing mechanisms are usually targeted with specific cell therapy approaches.

Although the implantation of cells has been associated with a significant enhancement in cognition, memory, and restoration of plasticity, the regeneration of neural tissue can be strongly stimulated by different biomaterials that help to connect the implanted cells with the host for further maturation into pre-existing circuitry [108]. The mechanical properties of brain tissue are in general below 100 kPa with stiffness values that range from 90 to 230 kPa in the case of spinal cord [78]. In this regard, 2% hydrogels of SF have been found to encounter mechanical properties that are similar to those of the brain tissue [77].

The features of SF for the healing of various stages of neuronal damage can possibly be split into two categories. The first one is defined by the properties related to the amino acid sequence and the by-products generated after SF degradation, which show antioxidant and anti-inflammatory properties. The latter is related to the biomaterial properties by itself (scaffold structure and biomechanical features) to interact with the target tissue providing tissue and cellular anchorage, stem cells, and/or drug delivery.

The in vitro evaluation of SF with primary culture of hippocampal neurons demonstrated the biocompatibility of SF fibers without cytotoxic effects [109]. The in vivo therapeutics of SF were inspected by Moisenvich et al. (2019). In a traumatic brain injury model in rats, they found that SF neuroprotected and restored the neurological status up to 25% after 4 days of injection, supporting the growth of primary neuronal cells and astrocytes without toxicity [110]. After cerebral ischemia, the silk hydrolysate showed a positive effect on visual and verbal memory [111].

The provision of stem cells alongside a biomaterial has been suggested as a regenerative medicine-based method that can lead to functional recovery. Although the invasiveness of intracerebral implantation of stem cells seeded with biomaterials for the usage in patients has been questioned, several clinical trials have reported positive outcomes after intracerebral implantation of stem cells, showing the feasibility of this approach to treat neural diseases [112,113,114,115].

In the context of stroke, our group designed an injectable silk hydrogel and assessed the quality and tolerability of the hydrogel after injection in the caudate putamen. It was demonstrated that the injection of silk hydrogels in the healthy mouse brain did not cause changes in the animal’s behavior in terms of sensorimotor skills, learning ability, or sleep–wake regulation [77]. Following this study, the biocompatibility of MSC encapsulated into silk hydrogels was confirmed in vitro [88]. Functional recovery associated with motor reorganization, similarly to what occurs in stroke monkeys and humans after rehabilitation, was reported by our group after the transplantation of silk fibroin and MSC [62,63]. In stroke mice, the intracerebral injection of silk hydrogels presented good biocompatibility within the cerebral cavity as a result of a high bioactivity and interaction with microglial cells, which leads to further regeneration [89]. Another important feature of the intracerebrally injected SF hydrogel is the lack of secondary inflammation [77,89]. While primary inflammation is mainly the result of ischemic damage, the secondary inflammation related to the interaction of the biomaterial and the surrounding tissue was not seen after SF injection [116]. The ability of SF hydrogels in combination with MSC to promote neuroprotection and functional recovery was confirmed in subsequent studies in a model of traumatic injury in the rat [79]. Yet the number of studies on this topic is limited and further experimental data can be beneficial for understanding the inflammatory response and cell and tissue remodeling after cell-encapsulated implantation.

## 5. Silk Fibroin in the Context of Neurodegenerative Diseases

Neurodegenerative diseases are conditions that cause progressive degeneration of neural networks. Neurodegenerative diseases such as Alzheimer’s disease (AD), Parkinson’s disease (PD) and multiple sclerosis (MS) frequently affect the elderly, and due to demographic changes, the social impact of these disorders is increasing. Such diseases can place people at risk of a wide range of disabilities or death [117]. Neurodegeneration is a form of progressive death, with a loss of structure and function of neural cells and is mainly characterized by an aggregation of malformed proteins inside intracellular and extracellular compartments, with an ultimate production of reactive oxygen species (ROS) and an activation of inflammatory signals.

Alzheimer’s disease causes progressive neurodegeneration and dementia mainly affecting those over 65 years old. Alzheimer’s disease was regarded as the sixth leading cause of mortality in the United States in 2019 [118]. The cause is largely unknown, although extracellular amyloid-β (Aβ), intracellular tau tangles, and a significant decrease in acetylcholine (ACh) content might contribute to inflammation, oxidative stress, and neuronal death in different brain areas, thus progressively deteriorating local and large neural networks [119].

Neural network disruption produces a wide variety of clinical signs, which profoundly affect cognitive abilities, especially the capacity to memorize sensory experiences, including verbal and or visual learning. However, neuronal damage and impaired neural circuitry can be reduced or healed by antioxidant agents and regeneration strategies. Anti-inflammatory agents, modulators of amyloid-β and tau pathways, stem cells, and biomaterials have been considered promising therapies.

The intrinsic properties of SF arising from the amino acid sequence can be used not only in the treatment of cerebral ischemia, but also in the context of Alzheimer’s therapeutics. For example, the hydrolysate of silk fibroin can be achieved by enzymatic [120], chemical, and thermal treatment [121], and the generated by-products have shown neuroprotection against the apoptosis caused by amyloid-β, retarding the production of ROS and leading to the inhibition of apoptotic Caspase 3 enzyme [122]. The resulting SF peptides also have significant anti-inflammatory properties [123]. The SF hydrolysate improved the ACh concentration and cognitive response in rat specimens [124] and enhanced memorial activity in human volunteers [125]. The oral administration of the hydrolysate had a significant neuroprotective effect, enhancing visual and verbal memory improvement [126]. An enzyme-treated hydrolysate of SF was administrated orally in rats one week after cholinergic nerve injury. In this case, silk peptides were able to increase the amount of ACh followed by up-regulation of the *ChaT* gene, increasing the expression of choline acetyltransferase, an enzyme required for ACh synthesis [127].

Furthermore, the neuroprotective effect of SS protein was demonstrated in an Alzheimer-induced rat model, where a significant increase in the amount of ACh receptors ran in parallel to an improvement of cognition [124,128,129]. In addition, SF and SS are also able to block various key enzymes in the progression of the degenerative disease per se to control the further progress of the degeneration [122,130].

Parkinson’s disease (PD) is a neurodegenerative central disorder caused by the progressive death of dopaminergic neuronal cells, mostly in the substantia nigra, which translates into a reduction of dopamine content. In patients, PD causes motor symptoms such as tremor, rigidity, bradykinesia, muscle cramps, and dystonia or speech dysfunction, alongside non-motor symptoms such as sleep disruption, pain, impaired memory, and dementia [131]. The degeneration of dopaminergic receptors produces behavior and psychology impairments including anxiety and reduced openness to the environment [132]. The aggregation of levy bodies composed of abnormal α-synuclein has been seen in many patients.

The usage of L-DOPA, with the commercial name of Levodopa, as the precursor of dopamine, is the most common approach to increase the concentration of dopamine in PD patients [131]. L-DOPA can successfully activate dopaminergic neurons to produce more dopamine, reducing the motor and non-motor related side effects of the disease. However, the long-term treatment of PD patients with L-DOPA causes important disabilities related to the on–off phenomena, where dyskinesia and other abnormal motor fluctuations are very common, especially in patients that respond poorly to L-DOPA, and where, progressively, the periods of improved motility (ON) are very scarce in comparison with the periods of impaired motor function (OFF) [130]. In the brain, L-DOPA is found to have a reaction with iron and ascorbate, resulting in the creation of hydroxylated products of L-DOPA such as 6-hydroxydopamine (6-OHDA) [133], which is a recognized inducer of PD and is one of the common neurotoxins that could selectively damage the catecholaminergic nerves, including dopaminergic neurons [133,134]. Additionally, such unwanted sub reactions can cause further ROS generation in various pathways (reviewed by Hernandez-Baltazar et al., 2017 [134]). The production of ROS involves the massive destruction and dysfunction of dopaminergic neuronal cells [135]. Additionally, it has been found that PD is followed by the production of tyrosinases that are involved in neurodegeneration [136]. In PD, overexpression of tyrosinases causes hydroxylation, which results in a reduction in dopamine content and the production of ROS, which both cause neuronal cell death [136,137].

SS has been found as an inhibitor of tyrosinases [138], while the peptides of SF have shown neuroprotection against 6-OHDA-induced neurodegeneration [137]. SF-derived peptides preserved the viability of dopaminergic neurons in response to 6-hydroxydopamine neurotoxicity in a PD animal model induced by 6-OHDA [130]. Alternatively, it has been reported that SF peptides show inhibitory activity against monoamine oxidases (MAOs), important enzymes that cause the breakage of monoamines such as L-DOPA [137]. Extensive activity of silkworm extracts against MAO-A and MAO-B, in areas of the substantia nigra and cerebral cortex, was reported [47], and this concept was extended in a second work, where the injection of silk extracts antagonized the effects of *N*-methyl-4-phenyl-1,2,3,6-tetrahydropyridine, a precursor of the neurotoxin 1-methyl-4-phenylpyridinium, which is involved in the destruction of dopaminergic receptors within the substantia nigra [46]. Tyrosine content is reduced significantly after L-DOPA therapy and is a substrate of the tyrosine hydroxylase, an enzyme that presents reduced activity in PD and is regarded as an early marker of PD.

SF has shown competing results for the delivery of tyrosine in PD rat models [130,139]. In Kim et al. (2011), tyrosine was delivered from silk fibroin administrated orally, reducing the production of 6-OHDA and neuronal loss, with improvement of memory capacities in a PD rat model [130]. Stem cell-based therapies are also very promising approaches to reconstructing the injured substantia nigra-striatal pathway in Parkinson’s disease [140,141]. The increasing survival of transplanted dopaminergic cells has been demonstrated with several materials, such as collagen, poly(l-lactic acid)/xyloglucan, agarose and Arg-Ala-Asp-Arg self-assembled hydrogels (RADA hydrogels) [63].

Based on the biocompatibility of the SF biomaterials in vitro and in vivo and the properties of this material that drive neuronal migration and axonal guidance [53], it would be interesting to examine whether SF might have a significant application for axonal tracts growth and the functional rewiring of localized neurodegenerative damages.

## 6. The Importance of Silk in Peripheral Nerve Injury

A main cause of localized PNS traumatic injuries is severing of one or more nerves produced by an external force or trauma. In this case, neurons lost their connections and will need a pattern for regeneration. In general terms, and depending on location and extension of damage, PNS injury is more salvable than CNS damage. Although the regenerative capacity of peripheral nerves is potentially plausible, many patients show permanent motor and sensorial deficits.

**Figure 6:**
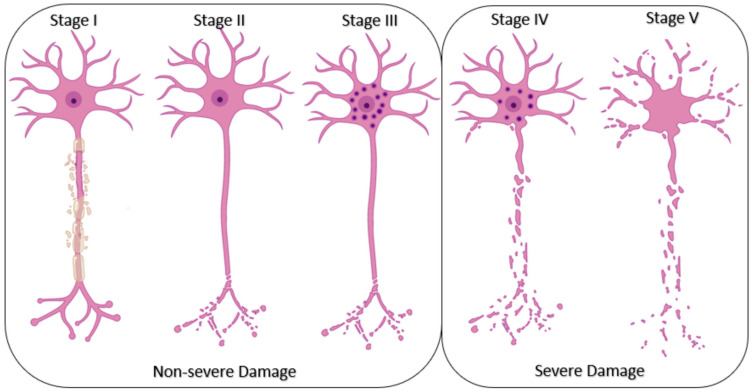
The stages of peripheral nerve cell damage. The lowest grade of damage starts with the degeneration of myelin bundles and it is regarded as the first grade. Second grade is characterized by the degeneration of neuronal axons and intact stroma while in grade III the disruption in the cell content and funiculi is visualized in the nerve bundles. In grade IV degeneration affects the whole cell and in grade V, the damage includes the neuronal trunk.

Following Seddon and Sunderland’s works, PNS damage can be divided into five categories based on the extension and severity of the injury [142,143,144] (Figure 6). Grade I to III damages are regarded as a non-severe injury where neurons still have the ability to self-heal using their intrinsic regeneration potential. This type of damage might be cured by exercise, physical therapy, and mild treatment approaches [145]. However, grades IV and V need complex medical therapies and allografts/autografts surgery [146]. End-to-end neurorrhaphy is only possible for short length gaps (<10mm) and nerve autografting is limited by tissue availability. By contrast, a limitation of most allografts is the use of extensive immunosuppression methods up to 18 months after the implant that exposes the patient to opportunistic infections [147]. Usually, 5 years after regeneration, only 25% of the motor function and up to 3% of the sensory function are recovered [146]. Another approach is to connect the severed nerves using a conduit, the two main problems being related to the lack of biodegradation of the conduit biomaterial, that can triggers the inflammatory response and the necessity of a second operation for removing the biomaterial [148], and to the intermingled of sensory and motor fibers that leads to erroneous re-innervation of the target tissue [149].

Silk fibers might address these two limitations [149]. The biodegradable and biocompatible properties of silk can be exploited to create conduits to join cut nerves, stabilizing the two cut edges of the nerve and stimulating growth thanks to the ideal elasticity, tensile strength, and tear strength. Silk fibers encounter a significant potential for the differentiation of stem cells into neural phenotypes [150]. A set of studies regarding the application of silk fibers as neuronal conduits was conducted by Gu et al. [151,152,153]. The initial study for the assessment of silk fibers as conduits in the PNS was done by seeding the rat dorsal root ganglia alongside Schwann cells [151]. It was reported that axons were able to grow in the direction of the microfibers covered with Schwann cells after 21 days of cell culture [151]. Seeded cells were viable, and silk fibroin did not exert any cytotoxicity with positive regeneration of the peripheral nerve [151]. The in vivo experimentation of PNS injury is mostly done in a sciatic nerve model where most of the autografts can bridge at most 5mm of the gap between the sciatic nerve and there is a need for an allograft once the damage is more progressed. In this regard, the biodegradation, biocompatibility, and inertness of the applied allografts are very important for PNS conduits, and SF has the potential to satisfy these conditions.

Non-functionalized electrospun tubular silk mats have been used as a conduit to regenerate PNS in a sciatic nerve-damaged rat model [55]. The study reports successful results for bridging a 10 mm gap within the sciatic nerve [55]. The conceptual framework of this study was confirmed in a more recent study, and similar positive incomes were observed [99]. In line with these results, Gu et al. assessed the ability of SF fibers with chitosan for providing a neuronal-like niche for culturing Schwann cells that were able to deposit ECM in the porosity of the biomaterial [152,153]. The combination of chitosan–SF fibers and the secreted ECM has been used to successfully fill 10 mm gaps [152]. Similar results were achieved with bone-derived MSC cultured in a chitosan–SF fiber composite [153].

Following the successful results with SF fibers, the regeneration properties of the 30 mm gap filler silk fibroin scaffolds have been assessed by Xue et al. in the sciatic nerve damage inside of the dog. This model resulted in a similar quality of post-damage regeneration than in autologous nerve grafts [154].

## 7. Increasing the Performance of Silk Fibroin in Neural Tissue Engineering

The natural properties of silk can be also further modified to improve the quality of cell adhesion, induce polarity to the biomaterial, and further tune the bulk and the surface properties. Some of such improvements in the biomaterial have been also applied in the context of drug delivery. In this section, we describe such strategies in the arena of SF usage.

### 7.1. Improvement of Cell Adhesion

Through different sets of studies in various formats of SF, it has been confirmed that SF can offer minimal requirements for cell adhesion, which is essential for cell integration and regeneration [62,78,88,90,91]. SF itself has been used as a coating to improve the osteoblast and the fibroblast biomaterial interaction as well as for alloys coating in biocompatible stents [155,156,157]. However, several points can be targeted for further improving the SF performance. ECM is a general aspect of cell biology that significantly controls the cell survival, migration, proliferation, and maturation and interaction with cell–adhesion proteins is important for cell contact and communication with the ECM compartment [91]. The cell attachment properties of SF biomaterials can be further improved by refining the residual negative charge of silk fibers [61]. Due to the lack of bioactive molecules in SF, the functionalization of this biomaterial with ECM proteins (such as fibronectin, laminin and collagen), ECM-adhesive peptides (such as the peptide sequences Ile-Lys-Val-Ala-Val; IKVAV and Arg-Gly-Asp; RGD) and growth factors (such as nerve growth factor and fibroblast growth factor [100]) have been applied in numerous studies.

Coating SF biomaterials with positive-charge-based polymers is a good strategy to modify the partial charge of SF, enhancing the adhesion of stem cells and neuronal progenitors promoting neuronal outgrowth by interaction with the negatively charged surface of the cells. Polymers such as polylysine, propylenemeine, and polyethyleneimine (PEI) have been conventionally used for neuronal cell attachment [158]. Perhaps one of the most interesting strategies is the application of reactive polycatechol for the stabilization of neuronal cells [158,159]. Polycatechol can react with serum proteins to make a coating of the proteins on the biomaterial and form a connection to the cells [158].

Coating the biomaterial with ECM proteins might result in the provision of binding domains for adhesion receptors on the cell membrane, thus increasing cell adhesion and performance of the local biomechanical forces to cells [160]. Molecules such as laminin, fibronectin, and collagen are examples of such ECM proteins. Perhaps one of the most relevant composites for improving the bioactivity of SF is based on collagen–silk formulations. Due to the high number of bioactive molecules in collagen, the application of collagen compensates the lack of cell-adhesion motifs in the SF [158]. For example, in the Kaplan’s cortical brain model, collagen–fibroin composites were decorated with Poly-D-Lysine, which significantly increased the attachment of neuronal cells [60]. The integration of collagen hydrogels within silk sponges showed promising results in modeling 3D polarized gray matter in the neuronal tissue [59] and cortical brain [60]. Another application of collagen/SF is found in spinal cord injury models, where this composite sustains NSC successfully and induces regeneration [57].

Other commonly used ECM-based proteins for coating silk fibroin are laminins. Laminins are composed of various α, β, and γ chains and are strongly bioactive molecules found in the basal lamina with an important role in cellular adhesion and differentiation. In silk fibers, the treatment with laminin has been found to promote cell migration, proliferation, nerve regeneration, and neurite outgrowth with considerable axonal extensions [100]. The laminin on the surface of electrospun SF mats has been coated to improve the proliferation, differentiation, and survival of neuronal progenitor cells [161]. Furthermore, the implantation of laminin/silk protein was able to enhance regeneration in a hemisection spinal cord defect in a rat model [162].

The ECM proteins contain one or several short peptide motifs involved in several cellular processes such as cell adhesion, migration, and proliferation. The natural SF from various species of insects such as *Antheraea yamamai* and *Antheraea pernyi* has various RGD sites with the ability to improve cell adhesion, while the RGD content in the *B. mori* (silkworm) is lower [163]. Consequently, the integration of more informational molecules within the silkworm SF sequence is a strategy for increasing the performance of the silk fibroin. Kang et al. (2018) have produced recombinant SF films with RGD sequences integrated in the cloned gene of *B. mori* [163]. Furthermore, the peptide sequence IKVAV derived from laminin has shown a significant impact on the improvement of neurite growth and cell adhesion. The potential of silk hydrogels for sustaining NSC has been demonstrated by the successful encapsulation of NSC within IKVAV-functionalized silk hydrogel, and good viability was shown at one week after NSC internalization, with clear signs of neuronal differentiation [90]. This study confirms the fact that SF coating increases cell attachment, survival, and maturation, and highlights the particular contribution of silk-fibroin-based hydrogels in soft tissue engineering.

### 7.2. Growth Factors and Drug Delivery

Growth factors (GFs) are included in the family of trophic factors and are naturally occurring molecules capable of binding the cells to regulate many aspects of cellular function. Several GFs such as nerve growth factor (NGF) and fibroblast growth factor (FGF) are normally secreted from glia and neuronal cells in response to trauma conditions in a bell-shaped trend with the highest amount in the early phases of the injury and the reduction within one month of the damage [145,164]. The reduction in GFs secretion causes a significant decrease in the neuronal regenerative ability [145]. Consequently, after neural damage, the supplementation with extra exogenous GFs might help to enhance the regeneration process beyond the limited endogenous capacity.

GFs are heat-sensible proteins subject to proteolytic degradation with a short half-life. In the context of nervous system disorders, the integration of neural GFs with silk-based biomaterials has been associated with positive outcomes mostly due to the capacity of SF, like other biomaterials, to provide a continuous and controllable source of GFs as a result of the progressive biodegradability of the biomaterial [78]. Additionally, loading the GFs on silk fibroin has other advantages, such as providing a targeted administration for the regeneration [145], providing a better niche for neuronal cells, and protecting the integrated GFs from the enzymatic activity of the host environment. Coating the biomaterials with NGF, an essential neuronal growth factor for neurotrophic function and regeneration, is a popular strategy in nerve tissue engineering [22,68,106]. Since several GFs are secreted during the process of natural regeneration, loading only one growth factor to the biomaterial might not be sufficient for best results. Thus, what is usually studied is the effect of a combination of two or more GFs exposures to the damaged tissue. Additional GFs such as Glial Cell-Derived Neurotrophic Factor (GDNF) and Ciliary Neurotrophic Factor (CNTF) have been used in combination with SF and have shown a desirable complementary effect on the regeneration of the neuronal damages [68,106]. It has been found that growth factors such as NGF and CNTF can produce an extensive neuroprotective effect, inducing neuronal remyelination and survival [165,166].

Based on the biodegradation properties of SF, it is possible to direct the delivery of GFs by integrating them within the bulk or the surface of the biomaterial. For example, the Vascular Endothelial Growth Factor (VEGF) and Brain-Derived Neurotrophic Factor (BDNF) have been integrated respectively within the surface or the core of the silk fibers. It was shown that, while the coating of VEGF on the surface and BDNF in the core has been linked with vascularization and neuronal regeneration of cavernous nerve, the integration of BDNF in the surface and VEGF in the center stimulated Schwann cell proliferation [167].

Table 2 illustrates examples of therapeutics based on the combination of SF and GFs for neural tissue engineering. In this topic, it would be interesting to examine the potentiality of SF to deliver other neurotrophic and angiogenic factors, such as insulin-like growth factor (IGF), neurotrophin-3 (NT-3) and fibroblast growth factors such as FGF1, FGF2, and FGF21, which have been used in non-silk fibroin platforms and have shown promising results [145].

By several techniques of coating and particle modification, SF can be loaded with small drugs to target specific areas. This will increase the bioavailability and will reduce the effective dose of the required drug. However, in the context of CNS disorders, an important challenge is the presence of the BBB, which limits the passage of substances administrated systemically. The delivery of drugs to the brain becomes even further challenging in injured brains, e.g., due to a traumatic injury or brain edema, as the result of damage limits the drug diffusion significantly [177]. Although a breach in the BBB permeability occurs after injury [178], systemically administered molecules might not necessarily have open access to the brain.

To overcome these challenges, different silk formats such as films and micro- and nanoparticles can be applied. For example, SF films have been used to bypass the BBB to deliver small molecules that reached deep brain structures [35]. The particular properties of SF make this biomaterial ideal for coating nanoliposomes on targeted drug delivery [33]. However, the utilization of SF microparticles directly or in combination with other biomaterial particles has not been taken into profound consideration.

The potential of SF microparticles as a biocompatible regenerative construct has been discussed and assessed by Moisenovich et al. (2019). They found that the delivery of SF and SF/gelatin microparticles did not cause any toxicity or activation of microglia and astrocytes. Similarly, liposomes have been reported as efficient nanoparticles to bypass the BBB. Additionally, liposomes have been found to improve the therapeutic index, increasing the drug half-life and reducing side effects [34], and SF has been utilized as a coating for enhancing biocompatibility and integration of the target surface molecules.

Drug delivery with silk fibroin and their promising results in non-neuronal tissues has been reviewed by Pham et al. [179]. The concept of drug delivery using silk nanoparticles (particularly curcumin-loaded silk fibroin nanoparticles) has been done in other areas of tissue engineering such as cancer therapy [180] and wound healing [181] with anti-microbial, anti-oxidant and anti-inflammatory properties [180].

The application of silk nanoparticles to bypass the BBB might constitute an attractive paradigm to neuroprotect and regenerate the damaged brain after trauma or neurodegeneration. An interesting example of silk for drug delivery can be considered for the treatment of epileptic seizures. Adenosine augmentation therapies have been found as an effective approach to suppress epileptic seizures based on the anti-convulsion effects of this nucleotide [182,183]. Systemic administration of adenosine has been associated with significant cardiovascular effects [184,185]. Thus, a local delivery of this drug is preferable to enhance safety in epileptic patients. SF was used to sustainably release up to 1000 ng/day adenosine to the rat brain tissue 48 h after implantation of the biomaterial in the intra-hippocampal cleft (intra-cerebral injection) [185]. Following this study, the efficacy of silk films as a delivery method of adenosine has been assessed in the kindled rat model showing anti-ictogenic and neuroprotective effects [186]. The adenosine delivery approach via silk films has been registered as a patent with therapeutic potential in the treatment of epilepsy [187].

### 7.3. Tuning the Biomechanical Properties and Degradation

By creating composites of SF and other materials, it is possible to tune the mechanical and bulk properties of the material and enhance the attachment of cells to the fibers. For example, the mixture of cellulose fibers and SF sponges were linked with extensive cell attachment potential. The cellulose fibers have a partial solubility upon the addition of lithium bromide solution so the SF/cellulose composite can be created by mixing these solutions in 9.2 M LiBr [80]. It was also stated that the rate of biomaterial biodegradation was dependent on cellulose fiber concentration [80].

Composites of PLA/SF have been spun by electrospinning with an integrated growth factor (NGF) that was able to sustain the attachment and growth of neural cell lines [168]. In this study, while SF was utilized to integrate NGF, PLA was added for an adjustment of the degradation and controlled release of NGF [168]. The efficiency of SF to enhance the delivery of NGF from hydroxyapatite with respect to formulations based on bovine serum albumin and hydroxyapatite has also been reported [188].

Table 2 generalizes examples of studies with SF composites in the context of neural tissue engineering.

### 7.4. Providing Alignment and Polarity

Providing a polar pathway is important for guiding neuronal cells, further maturation, and differentiation. Composites of polar spun and non-polar biomaterial can provide an arrangement in non-polar 3D silk biomaterials and scaffolds such as sponges and hydrogels [102]. As a result of the biomechanical tension fibers will compensate for the lack of arrangement and polarity of non-fiber SF 3D materials [168]. Composites of SF–carbon nanotubes can provide a suitable guide for neuronal cells and act as a conduit for axonal reconstruction and neural regeneration [22,189].

Performance of the physical surface modifications to the biomaterial and the alteration of the topology by patterns or molecules can introduce polarity and arrangement of the biomaterial to guide proliferation and cellular migration. Techniques such as photolithography, pattern stamping and lyophilization have been utilized to provide polarity and patterns for cells guiding [190,191]. The biomechanical properties of SF, the ease of modification, and the surface wettability have made this material a good platform for microfluid-related investigations [192]. For example, multiple uniaxial microchannels (42–142 mm) have been engraved in silk hydrogels [191]. Following the seeding of hippocampal neurons, it was found that the cells could follow the direction of the engraved micro-ridges and started the establishment of cell-to-cell and cell-to-matrix interactions [191]. It was also found that the ridges in the inner surface had a role in the neuronal extension and adhesion by the provision of guidance [191]. For the alignment of cells, the size of the micropatterns and the mechanical properties are important. Recently, the effect of the topography of the surface and the bulk concentration of the SF biomaterials have been assessed in SF films, with an obvious nerve growth within grooves of a 30 μm width patterned within SF films 15% concentration [190].

Another strategy to guide cells is to establish topological patterns by coating them with bioactive molecules. One of the first studies in this regard was done by Kang et al. (2004); where the gradient dispensing with RGD molecules caused a significant arrangement of fibroblast cells [193]. This strategy has been extended to neuronal cell tissue cultures, where the gradient and anisotropy of laminin molecules stimulated neuronal alignment and cell recruit [194]. Other studies confirmed the orientated growth pattern of neuronal cells and better functional recovery of peripheral nerve gaps in the presence of gradients of neuronal growth factors such as NGF or laminin-1 [195,196]. These studies introduced the idea of ingredient dispensing and the usage of SF biomaterials. Tropoelastin-coated silk films patterned by 3.5 μm grooves showed a directional outgrowth of neuronal and Schwan cells [174]. Dinis et al. reported a method for the fabrication of electrospun fibers, showing that the application of the gradient and functionalization with NGF and CNTF could increase the orientated growth and neurite outgrowth of DRG neurons [106]. This seminal study can be an introduction for the usage of a gradient of active biomolecules for the provision of orientation of neural cell lineages.

Recently, we have identified that SF fibers obtained through SFS seem to have a natural compatibility for axonal guidance [53]. Application of SF surface modification can be further improved once an external stimulus such as electrical stimulation is also introduced. This stimulus can be applied not only to trigger the release of a drug or GF [197] but also to increase the cellular arrangement and neurite outgrowth. The performance of an electrical stimulus regime to neuronal cells seeded within SF hydrogels [198] and films [64] has been found to significantly induce neurite outgrowth and alignment. Electrical stimulation has been also assessed in vivo by implantation of electrodes integrated into uniaxial multi-grooved silk films. This latter approach evoked neural calcium waves with minimal inflammatory response [65].

The application of mechanotransduction and intermittent mechanical stress regimes in neuronal outgrowth and maturation was found to be important in neural engineering [199], but external mechanical force and biophysical external force regimes (such as intermittent peaks and sinusoid waves of force patterns) in presence of SF biomaterials have rarely been studied. Additionally, light stimulation is another element that has been shown to be beneficial for guiding neurons [200]. A production method for the modification of SF films has been patented by Prof. Kaplan’s group for biophotonic and biomedical applications [201]. The integration of light stimulus with neuronal SF microfluidics or SF-based biomaterials might be another step toward the creation of further arrangements of maturated neuronal cells.

## 8. Concluding Remarks

It is only recently that we have realized the opportunities that silk affords in treating a variety of neurological disorders such as Alzheimer’s disease, Parkinson’s disease, stroke, and peripheral nerve injury. This natural material shows innate anti-inflammatory and neuroprotective properties.

It is relatively easy to structure silk proteins in various 2D and 3D formats including hydrogels, films, sponges, and fibers. The ultimate biocompatibility, biodegradability, and the ease of SF handling allow for a loading of stem cells, bioactive molecules, and other drugs to boost bioactivity and nervous tissue regeneration.

Despite the recognized advantages of silk fibroin in vitro and in vivo, the transition of the silk application from the scientific research to the large-scale industrial purpose is dependent on the further research to increase the reproducibility of the inter and intra batch utilization to produce a standard strategy that is resource-efficient, environment friendly, time efficient and cheap. At the same time the processing procedure should be endowed with the ability of automation and production of high-quality silk-based biomaterials. In this regard, the industrial production line of silk sutures (that are frequently used in neurosurgery; e.g., in cranioplasty for re-securing the flap bone or in dural closure to minimize postoperative cerebrospinal fluid leakage) reflect the range of properties offered by this material and how they can be combined with an industrial production [202,203,204,205]. Some more recent examples, such as the fabrication of coatings for breast implants [206] show the enormous opportunities offered by applying regenerated silk formats in the development of new therapies.

Another important point to focus, is the need for further experimentation to prove the usability of SF in the clinical area and the context of regulatory agencies. In this sense, it is essential to test the safety, tolerability, and effectiveness of different therapeutic SF-based products before their translation into clinics [207].

Addressing this problem, our group has performed a set of studies for the assessment of the safety and tolerability of SF in a hydrogel format in the mouse brain [77], which showed promising results in terms of the enhancement of the regeneration and remodeling of peri-lesional areas in a stroke model [62]. Another attempt to prove the safety and efficacy of SF was made in 2018 with an SF-based biomaterial called SilkBridge for bridging the PNS nerve gap [208,209]. In addition, two sets of clinical studies regarding the application of SF implants for the regeneration of vocal fold paralysis have recently been conducted [210,211]. Such present and future clinical studies are advantageous and will allow for further improvement in efforts to meet the required clinical conditions to treat neurological disorders.

## Figures and Tables

**Table 1:** Common methods of silk fibroin (SF)-based biomaterial production in neural tissue engineering.

Biomaterial	Fabrication Method	Main Applications	Advantages	Limitations
Fibers	Straining Flow Spinning [52,53]	Conduits for nerves	Easy instrumental method, easy bulk and surface property modification, high-performance individual fibers	Thick diameter of fibers
Mats, meshes, bundles	Electrospinning [54,55,56]	Conduits for PNS damages, spinal cord injury	Fabrication of ultra-fine nanometric fibers, control of fiber properties (orientation, diameter, and composition)	Usage of organic solvents, complexity in the control of various parameters and variability
Custom Shapes	3D printing [57,58]	Conduits for PNS damage, spinal cord injury	Highly reproducible, fabrication of complex 3D structures, integration with various polymers and/or cells	The nozzle and cartridges can affect cell viability, costly
Sponges	Solvent Casting/Porogen leaching [59,60]	Modeling polarized neural tissue Bioengineering of cortical brain tissue	Uncomplicated and easy-to-use instrumentation, coating of different biomolecules, low cost	Lack of ability to control pore communication and interpore channels
	Lyophilization [61]	Traumatic neural tissue damage	Control the mechanical and degradation properties by initial concentration, pH, and freezing rate	Extra treatments for β-sheet-enriched conformation
Hydrogels	Self-assembly [62,63]	Intracerebral applications, drug and cell delivery	No need for organic solvents or further treatment steps for induction of beta-sheet, production of ultra-fine microfibers	Complicated to define the self-assembly process, requirement of physical-chemical agents (e.g., sonication, cross-linkers) to induce gelation
Films	Dry Casting [35,64,65]	Drug delivery, axonal growth and guidance, neural electrodes covering	Relatively simple and low cost method, low invasiveness	Formation of films in silk I stage, necessity for further steps of treatment for induction of beta-sheet content

**Table 2:** Types of silk fibroin (SF) composites for neural tissue engineering.

Silk Fibroin Formulated with Synthetic Materials			
Target Cell/Tissue, In Vivo Model	Formulation	Main Results	References
PC12 (neural cell line)	SF/Polylactic Acid (PLA)	Elongated neurites (~95 μm), support cell attachment and differentiation	[168]
MSC	SF/Carbon nanotubes	Trans-differentiation towards neural cells	[169]
Schwann cells	SF/Gold nanofibers	Cell adhesion without toxic or immunogenic response	[170]
Schwann cells	SF/Graphene	Cell growth in an electroconductive and biocompatible surface	[171]
Neuronal progenitor cells/rat sciatic nerve model	SF/Carbon nanofibers (CNFs)/Poly-ε-caprolactone (PCL)	Cell-to-cell communication, regeneration of sciatic nerve model (~2 cm)	[172]
Schwann cells	SF/Polypyrrole	Arrangement of cells without toxicity	[58]
**Silk Fibroin Formulated with Natural Materials or ECM-Derived Peptides**			
**Target Cell, Tissue, In Vivo Model**	**Formulation**	**Main Results**	**References**
Sciatic nerve injury model	SF/Chitosan	10 mm nerve gap model bridging	[152]
Neuroblastoma cell line (SH-SY5Y)	SF/Melanin	Significant antioxidant potential, cell differentiation	[173]
NSC/rat spinal cord injury	SF/Collagen	Increasing nerve regeneration	[57]
Schwann cells	Silk/Tropoelastin	Cell arrangement and neurite guidance	[174]
MSC	SF/YIGSR and GYIGSR Integrin-binding laminin peptide motifs	Enhanced cell proliferation and differentiation	[93]
NSC	SF/IKVAV Integrin-binding laminin peptide motif	Improvement of cellular differentiation and viability	[90]
PC12/rat sciatic nerve model	SF/SF16 peptides	Enhanced cell viability and axonal growth	[175]
Hippocampal neurons	Silk/Laminin	Stimulation of cell growth, differentiation, and neurite extension	[162]
**Silk Fibroin Formulated with Growth Factors**			
**Target Cell, Tissue, In Vivo Model**	**Formulation**	**Main Results**	**References**
PC12	SF/PLA/NGF	Sustained release of NGF, increased neurite outgrowth (~95 μm)	[168]
Rat dorsal root ganglion neurons (DRG)	SF/NGF (gradient distribution)	Cell growth and orientation (NGF gradient)	[176]
DRG	SF/NGF/CNTF	Enhancement of neurite outgrowth	[106]
Schwann Cells	SF/BDNF/VEGF	Improvement of cell growth and vascularization	[167]

## Data Availability

Not applicable.
